# SPE-UHPLC-FLD Method for the Simultaneous Determination of Five Anthraquinones in Human Urine Using Mixed-Mode Bis(tetraoxacalix[2]arene[2]triazine) Modified Silica as Sorbent

**DOI:** 10.1155/2017/1963908

**Published:** 2017-09-28

**Authors:** Kai Hu, Yonghui Qiao, Zhifen Deng, Mingxia Wu, Wei Liu

**Affiliations:** ^1^Henan University of Chinese Medicine, Zhengzhou 450008, China; ^2^College of Chemistry and Molecular Engineering, Zhengzhou University, Zhengzhou 450052, China

## Abstract

The five anthraquinones compounds (including aloe-emodin, emodin, physcion, chrysophanol, and rhein) are regarded as the main effective ingredients in rhubarb (Dahuang in Chinese, one of the commonly used Chinese herbal medicines). In this work, a simple and effective solid phase extraction (SPE) method based on bis(tetraoxacalix[2]arene[2]triazine) modified silica gel as adsorbent was developed. Coupled with UHPLC-FLD, the developed method was successfully applied for the measuring of main anthraquinones in human urine after oral administration of the extracts of rhubarb. To obtain the highest recoveries of the five anthraquinones in the SPE process, the main parameters which may affect extraction efficiency were optimized. The optimized sorbent amount, sample loading pH, sample loading rate, washing solution, and eluent condition were obtained. The developed method showed good linearity in 0.012–1.800 *μ*g mL^−1^ for the five anthraquinones with correlation coefficients more than 0.9993. The investigated LOD values ranged from 3.9 to 5.7 ng mL^−1^, while the LOQs were between 12.0 and 18.2 ng mL^−1^. The recoveries of the method were also investigated, which were in the range of 94.8–106.6%. The application of the mixed-mode SPE materials in the proposed method was feasible and simple, and suitable for the enrichment of anthraquinones in urine samples.

## 1. Introduction

As a well-known herbal medicine in China,* Rhubarb* has been used to treat diseases since ancient times. Among all constituents in rhubarb, the anthraquinones (including physcion, rhein, emodin, chrysophanol, and aloe-emodin) play a dominant role for the medicinal properties of rhubarb and are regarded as important active ingredients with extensive pharmacological effects such as antiviral [1], antibacterial [2], antifungal [3], antioxidant [4], antiath-erosclerotic [5], and anticancer activities [6]. Thus, the investigation on the qualitative and quantitative analysis of anthraquinones in body fluids (plasma, urine, etc.) is always a hot research area in pharmacokinetic study.

Because of the complexity of sample matrixes and the trace levels of anthraquinones in urine sample and other biological sample, there is an urgent need for developing a sensitive method for the determination of anthraquinone compounds. Until now, there are many methods that have been conducted for the determination of anthraquinone compounds, such as capillary zone electrophoresis (CZE) [7], capillary electrochromatography (CE) [8], thin layer chromatography (TLC) [9], and micellar electrokinetic chromatography (MEKC) [10, 11]. However, due to its easy operation, high accuracy, and wide applicability, HPLC is still most widely used among these techniques [12–18]. On the other hand, many extraction methods, such as decocting or refluxing extraction [19, 20], liquid–liquid extraction [21], and ultrasonic extraction [22], have been widely applied for the enrichment and cleanup of trace anthraquinones in the complex biological and herbs samples. However, some of these methods are usually time-consuming, solvents, or labor-intensive. Due to its simplicity and low solvent consumption, solid phase extraction (SPE) methods [8, 12, 13] have emerged as effective alternatives for sample preparation method. However, the traditional reversed phase or ion-exchange sorbents are usually showing indiscriminate adsorption effects, resulting in a large number of matrix interferences, which may seriously affect the accuracy of the measurement. Therefore, it is necessary to establish an effective sample preparation method for the analysis of anthraquinones compounds in complicated body fluids samples.

Calixarene is a typical representative of the third-generation host molecule following crown ether and cyclodextrin. By modifying them onto the surface of silica gel, calixarenes have been widely studied as solid phase extraction and stationary phase materials. Due to the peculiar structural characteristics of cavity-shape and benzene ring skeleton, these materials can offer varieties of interaction with analytes, such as hydrophobic, hydrogen bonding, *π*-*π*, as well as inclusion interaction [23–31]. Nowadays, heterocyclic calixarene appeared as a novel kind of supramolecular compound [32]. Being different from traditional calixarene, heterocyclic calixarene is usually constituted of benzene rings and triazine rings connected by O or NH groups, which can not only manipulate the cavity, but also improve their recognizing selectivity toward a great diversity of guest species. Recently, by modifying bis(tetraoxacalix[2]arene[2]triazine) onto the surface of silica gel, a new separation material was successfully used as stationary phase and solid extraction material [33, 34]. The new separation material was exhibiting multiple-interaction and mixed-mode separation mechanisms, and excellent extraction ability toward flavonoids compound [33, 34]. As anthraquinones have similar molecular structures with flavonoids compound, excellent extraction capacity toward anthraquinone compounds by using the same sorbents is anticipated. Thus, in this study, the new material was utilized as new solid phase extraction material for enrichment and cleanup of anthraquinone compounds in the human urine.

In the present study, a novel bis(tetraoxacalix[2]arene[2]triazine) sorbent (BTO-SPE) was employed to develop a high-efficiency and reliable sample preparation procedure for the analysis of anthraquinone compounds in the human urine. All the main factors affecting SPE process were optimized to obtain the high recoveries. Based on BTO-SPE sorbents as solid phase extraction materials, a UHPLC-FLD method was developed and evaluated. The analysis results showed that it was appropriate for the enrichment and cleanup of anthraquinone compounds in the urine and other complex biological samples.

## 2. Materials and Methods

### 2.1. Chemicals, Materials, and Solutions

**Figure 1:**
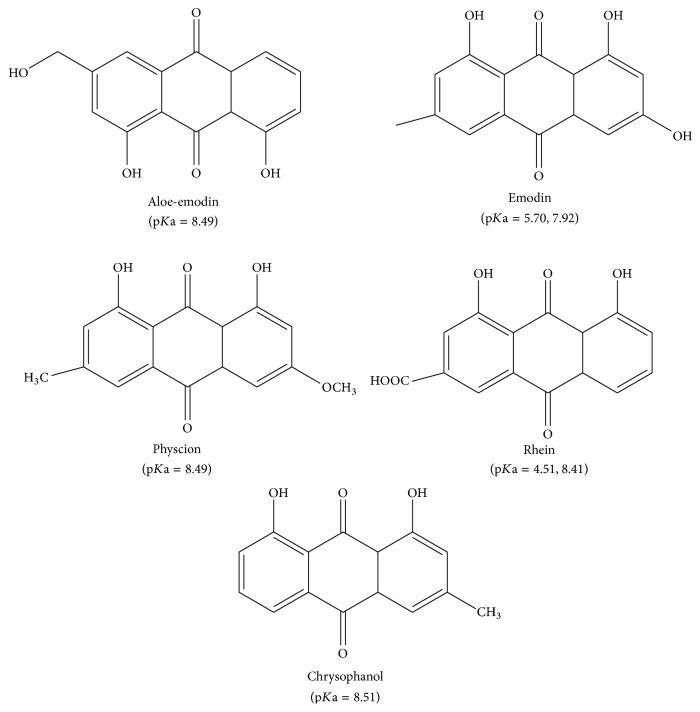
The chemical structures and p*K*a values of the studied anthraquinones.

The reference standards of chrysophanol, physcion, rhein, aloe-emodin, and emodin were provided by the National Institutes for Food and Drug Control (Beijing, China) (Figure 1). HPLC-grade acetonitrile, methanol (MeOH), and formic acid were purchased from Sigma-Aldrich Co. All other chemicals and reagents used were of analytical grade obtained from Shanghai Aladdin Chemical Reagent Company. The Polypropylene SPE tube (3 mL) used for packing the sorbent and the PE Frit (20 *μ*m) were all obtained from Shanghai ANPEL Laboratory Technologies. The water used for HPLC system and SPE process was prepared by using a Millipore Milli-Q system (Bedford, MA, USA). Silica gels (particle size of 40–60 *μ*m, surface area of 500 m^2^ g^−1^) were supplied by Shanghai Aladdin Chemical Reagent Company. The commercialized C18 SPE columns (100 mg, 3 mL) were provided by the company of Waters (Milford, MA, USA), which were used as comparison with self-made BTO-SPE.

By dissolving the reference standards into MeOH, the stock solutions of chrysophanol, rhein, physcion, aloe-emodin, and emodin were prepared at concentrations of 1 mg mL^−1^, respectively. Then, the stock solutions were mixed and diluted with 0.1% phosphoric acid water/methanol solution (50 : 50, v/v) to prepare working solutions. All the prepared stock solutions and working solutions were stored in a refrigerator at 4°C ready for use.

### 2.2. Instruments and Measurement

All separation was performed using an Agilent UHPLC configured with a binary pump (G4220B), a column compartment (G1316C), a FLD detector (G1321B), and an autosampler (G4226A). Elemental analysis was carried out on an EA 2400 elemental analyzer (PerkinElmer Corporation), and IR spectra were obtained by using a Nicolet iS5 FT-IR analyzer (Thermo Scientific). A centrifuge (Refrigerated Centrifuge XT, Thermo Scientific) and a Vortex mixer (Lab Dancer, IKA) were used for the centrifugal separation and thorough mixing of sample solutions.

The UHPLC chromatographic separation was carried out by using a Zorbax SB-C18 column (Agilent, 3.0 × 50 mm, 1.8 *μ*m) with mobile phase A (0.1% phosphoric acid water, v/v) and mobile phase B (MeOH) at the flow rate of 0.5 mL min^−1^. After careful optimization, the HPLC gradient eluent program was set. The detailed procedures were as follows: 0-1 min, 55% B; 1–4 min, 55%–70% B; 4–9 min, 70%–90% B; 9–9.5 min 90%–100% B; 9.5–10 min 100% B. The analysis temperature and injection volume were set at 30°C and 5 *μ*L, respectively. The excitation and emission wavelengths of fluorescence detection were set at 435 nm and 515 nm, respectively.

### 2.3. The Synthesis of BTO-SPE

**Figure 2:**
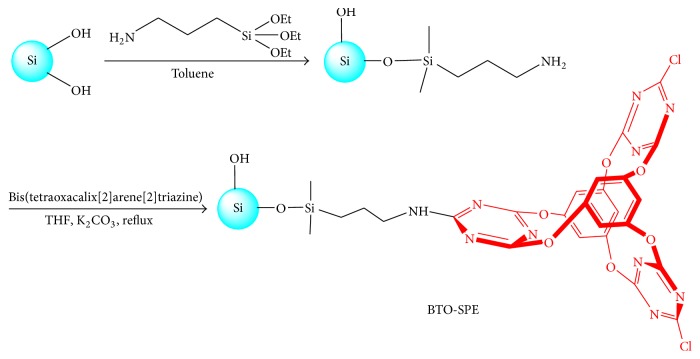
Preparation scheme of bis(tetraoxacalix[2]arene[2]triazine)-modified silica gel SPE sorbent (BTO-SPE).

The two-step preparation procedure of BTO-SPE sorbent was according the previous methods [33, 34] and illustrated in Figure 2. As can be seen from Figure 2, after 3-aminopropyl bonded silica gel (APS) was prepared; the mixture of APS, bis(tetraoxacalix[2]arene[2]triazine) and the reagent of tetrahydrofuran (anhydrous) were stirred in the stream of N_2_ for another 24 h. The final BTO-SPE was obtained by filtration through a G5 filter and washed by using methanol, water, and acetone.

### 2.4. Preparation of Urine Sample and SPE Procedures

Urine sample (1 mL) was piped into a centrifuge tube, and 1 mL MeOH was added. After being shaken quickly for 5 min, the above tube was then centrifuged at 12,000 rpm at 4°C for 15 min. After the supernatant was collected carefully, 1 mL 0.1% phosphoric acid was added to dilute the solution, mixed by a Vortex mixer, and the mixture was ready to load onto SPE cartridge for the further enrichment and purification.

BTO-SPE (100 mg) was accurate weighed and filled into an empty cartridge (3 mL); a PE Frit (20 *μ*m) was placed above and below the sorbent, respectively, to prevent the loss of sorbent. Then, the applicability of the prepared cartridge for the enrichment of anthraquinone in human urine was investigated. In brief, after the cartridge was activated by treatment with MeOH (3 mL) and water (3 mL), 1 mL treated urine sample spiked with analytes was flowed through the BTO-SPE cartridge by gravity. The SPE cartridge was carefully flushed by MeOH/water solution (2 mL, 5%, v/v) and eluted by methanol (2 mL). The eluent was evaporated to dryness at 35°C under a gentle N_2_ stream and then redissolved into 100 *μ*L 0.1% phosphoric acid water/methanol solution (50 : 50, v/v). After filtration through a 0.22 *μ*m Nylon filter (Waters, USA), the redissolved solution was ready for UHPLC-FLD analysis.

## 3. Results and Discussion

### 3.1. Characterization of BTO-SPE Sorbents

**Figure 3:**
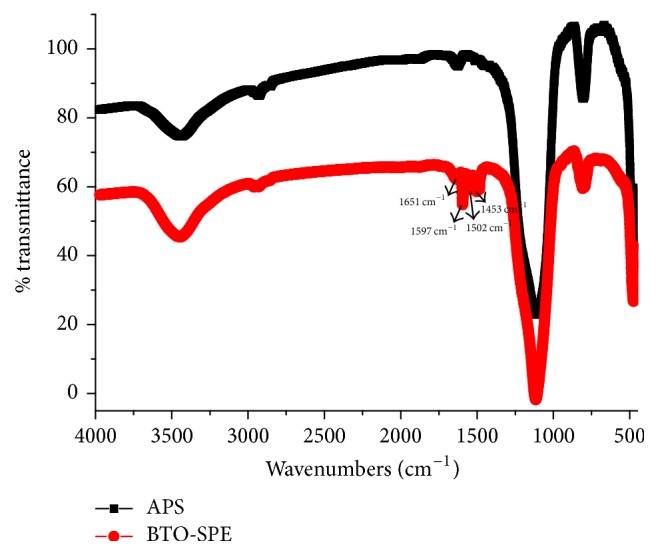
IR spectra of APS and BTO-SPE sorbent.

Infrared spectra of APS and BTO-SPE were shown in Figure 3. Compared with the two spectra, the new absorption peaks of 1597, 1502, and 1453 cm^−1^ were observed in BTO-SPE's spectrum, which may be all caused by the C-C stretch vibration of benzene ring skeleton. These significant differences indicate that the heterocyclic calixarene of bis(tetraoxacalix[2]arene[2]triazine) was successfully bonded onto the surface of microsphere.

Firstly, quantitative characterization of APS and BTO-SPE was conducted by the measure of elemental analysis. The content of C, H, and N between APS and BTO-SPE varied a lot. As the result showed, the C, H, and N in BTO-SPE were 12.87%, 1.38%, and 5.35%, respectively. The values were higher than those of APS (the content of C, H, and N accounted for 4.72%, 1.15%, and 1.29%, resp.), indicating bis(tetraoxacalix[2]arene[2]triazine) was successfully modified onto the surface of silica gel. According to the change of carbon content, the amount of bis(tetraoxacalix[2]arene[2]triazine) onto the surface of silica gel can be calculated as 323 *μ*mol g^−1^.

### 3.2. SPE Optimization

In this study, to achieve maximum adsorption efficiency in the SPE process, the effect of the main factors affecting the recoveries (*n* = 3) of anthraquinones was evaluated.

#### 3.2.1. The Amount of Sorbent

**Figure 4:**
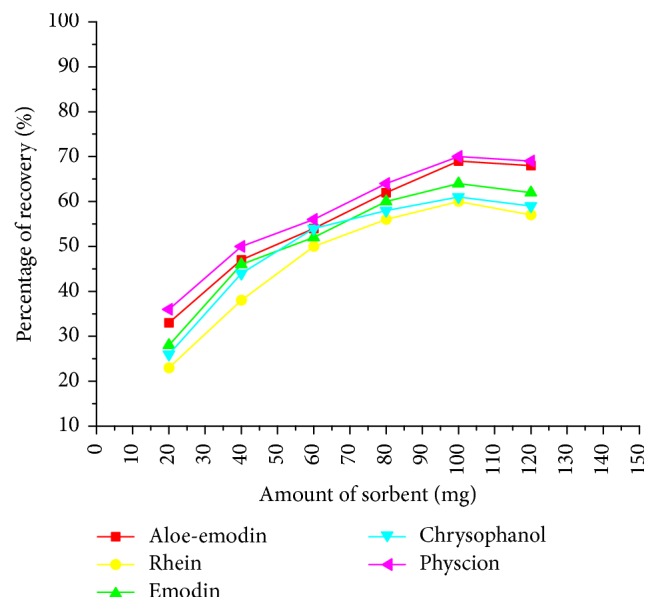
Effect of BTO-SPE sorbent amount on the recovery of anthraquinones.

The sorbent amount in the SPE cartridge is closely related to its purification effect. In this section, the influence of sorbent amount on the extraction recoveries of anthraquinones (1 mL working aqueous solution, 0.5 *μ*g mL^−1^) was studied. As can be seen from Figure 4, the recoveries of anthraquinones were increased with the amount of sorbent increased from 20 to 100 mg, and no significant difference of the recoveries was observed with the sorbent amount from 100 to 120 mg. Therefore, it can be inferred that 100 mg of BTO-SPE was sufficient to obtain satisfactory extraction recoveries and purification efficiency for anthraquinones. Thus, the optimal amount of the sorbent was selected as 100 mg in the study.

#### 3.2.2. The Effect of Sample Loading pH

**Figure 5:**
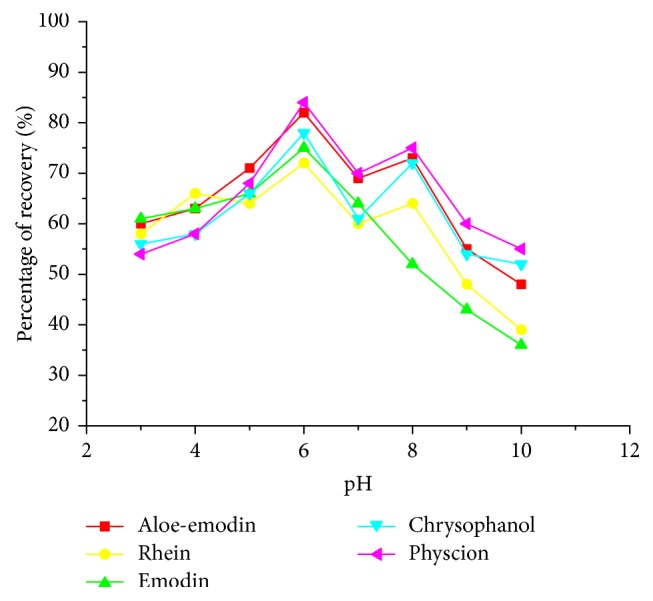
Effect of sample pH on the recovery of anthraquinones.

As anthraquinones have many hydroxyl groups, they may present in different states with the adjustment of pH medium; the SPE of anthraquinones probably seriously relies on the sample pH. Moreover, sample loading pH can also influence the solubility of the target acidic/basic solutes. Thus, in this section, the effect of sample loading pH (in the range of 3 to 10) on the extraction recoveries was studied. It can be observed from Figure 5 that the sample loading pH had a strong impact on the extraction recoveries of the target anthraquinones.

The highest extraction recoveries were obtained at pH 6, with the recoveries of 78%, 72%, 75%, 82%, and 84% for chrysophanol, rhein, emodin, aloe-emodin, and physcion, respectively. Therefore, pH 6 was selected as the optimal pH value for following extraction process. The reason for the optimized results can be attributed to the protonated or deprotonated states of anthraquinones compounds. As anthraquinones have many hydroxyl groups, they may exist in the form of protonated format in lower pH value and deprotonated format in basic pH solution. However, it is difficult for the reversed-phase material to strongly absorb the hydrophilic guest (protonated or deprotonated). Moreover, it can weaken the hydrogen bonding interaction between the target anthraquinones (protonated or deprotonated) and heterocyclic calixarene with the change of pH values in the sample loading solution. On the other hand, anion exchange interaction may also enhance the absorption interaction when the pH conditions were higher than those of the analytes' pKa values. Thus, the deprotonated rhein and emodin can have higher recoveries during the extraction procedure at pH 6. Therefore, the sample was adjusted to pH 6 in the following extraction process.

#### 3.2.3. Flow Rate of the Sample

The sample loading flow rate was an important factor that affected both the extraction recoveries and the sample preparation time. Under controlled negative pressures, the sample loading rate was optimized between 0.5 and 2 mL min^−1^. As a result, no distinct variance on the extraction recoveries of target anthraquinones was discovered by changing the flow rates of sample. As the volume of the sample was only 1 mL, it only took less than 2 min to pass through the SPE cartridge by gravity, so the sample loading rate almost has no effect on the extraction recoveries and extraction time. Thus, we did not control the sample loading rate and let the sample pass through the SPE cartridge by gravity.

#### 3.2.4. The Elution Step

**Figure 6:**
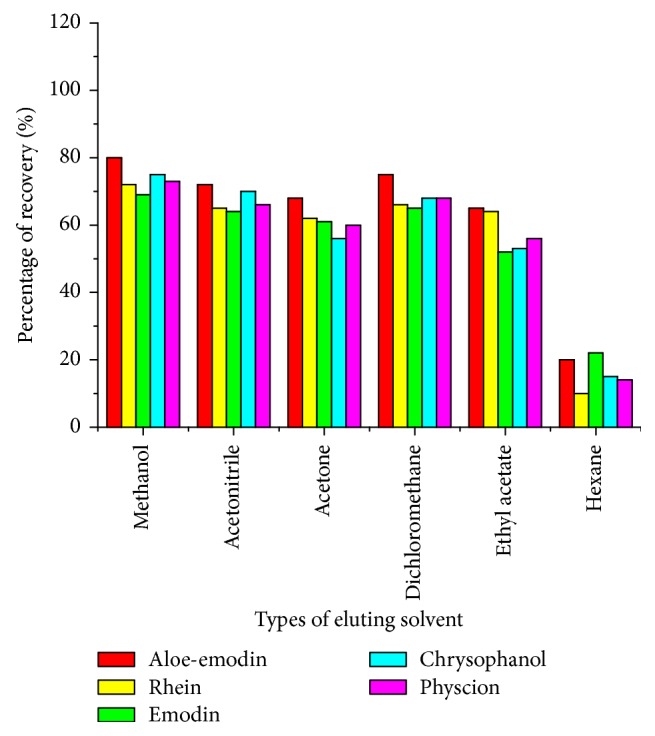
Effect of eluting solvent on recovery of anthraquinones.

In order to eluate the target analytes in the solid phase extraction process, it is of great importance to select a suitable eluting solution. In this section, six eluting solutions of different polarities, including methanol, acetone, acetonitrile, ethylacetate, dichloromethane, and hexane, were introduced to acquire the most appropriate elution solution. As shown in Figure 6, by using the polar elution solvents (such as methanol, acetonitrile, and acetone), high recoveries were obtained compared with that of nonpolar solvents (such as dichloromethane, hexane, and ethyl acetate), which may be attributed to the fact that elution solvents with strong polarities can displace the target analytes in very little volume from the sorbent. As can be seen from Figure 6, hexane showed poor elution capability toward all the studied anthraquinones, indicating BTO-SPE sorbent had strong adsorption capacity toward anthraquinones compounds. Moreover, among all the elution solvent, highest extraction recoveries for all the investigated anthraquinone compounds were acquired by using methanol. Thus, in the following extraction experiments, methanol was selected as the most suitable elution solution.

The volume of eluent was investigated in the range of 0.5–5 mL. With the volume of MeOH increased from 0.5 to 2 mL, the extraction recoveries of anthraquinones compounds increased. However, with the volume increased from 2 to 5 mL, the recoveries were slightly decreased. The possible reason can be ascribed to the dilution of sample and the loss in the process of nitrogen blowing process. Therefore, the volume of eluent of methanol was selected as 2 mL in the following extraction process.

#### 3.2.5. The Washing Step

The main aim of the washing step in the SPE process was to reduce interfering substances as much as possible and raise the recovery of the target analytes. In this study, the impact of washing solution (such as the composition and volume) on the extraction recoveries of target anthraquinones (1 mL urine sample spiked with 0.05 *μ*g mL^−1^ working aqueous solution) was investigated. As the main interfering compounds in the human urine were water-soluble matter, different concentrations of MeOH-water solution (2 mL) were employed to evaluate the effect on the recoveries of anthraquinones. The results indicated that the recoveries remained unchanged when the content of MeOH ranged from 2 to 5%. However, with the content of MeOH increasing from 5 to 20%, the extraction recoveries decreased gradually. To reduce interfering matter in the human urine as much as possible, 5% MeOH was selected as the optimum washing solution in the following SPE process. Then, the volume of washing solution was also investigated. The results showed that highest extraction recoveries were obtained by using 2 mL of 5% MeOH solution. Therefore, 2 mL 5% MeOH solution was employed to wash the sample in the extraction process.

#### 3.2.6. Comparison of SPE Cleanup between BTO-SPE and C18 Sorbent

In this section, the solid phase extraction performance of BTO-SPE sorbent with commonly available C18 cartridges was compared and investigated. To conduct the comparison, the C18 cartridges possessed the same specifications strictly as the that of homemade BTO-SPE cartridges, the SPE enrichment and cleanup procedures were manipulated as the steps previously described.

**Figure 7:**
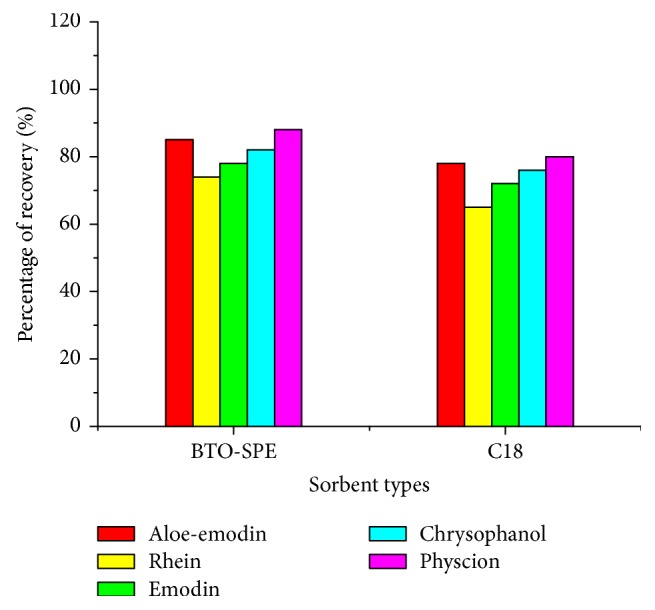
Comparison of the performance of BTO-SPE with C18 sorbent.

**Figure 8:**
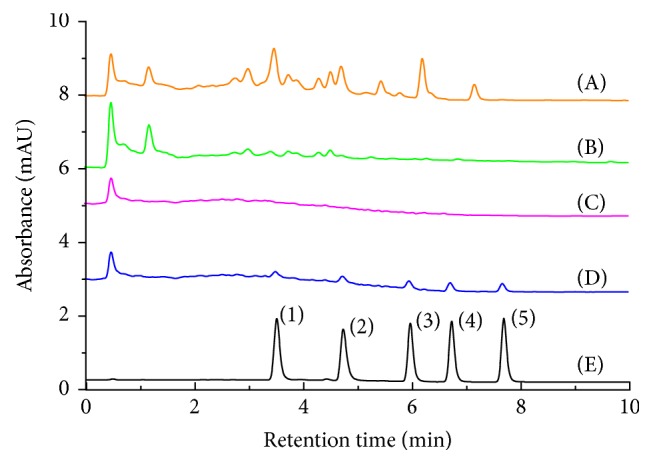
UHPLC-FLD chromatograms of samples and the standard solution: (A) blank urine sample without SPE cleanup; (B) blank urine sample after C18 SPE cleanup; (C) blank urine sample after BTO-SPE SPE cleanup; (D) urine sample spiked with 0.02 *μ*g mL^−1^ standard solution of five anthraquinones; (E) standard solution of five anthraquinones (0.5 *μ*g mL^−1^) (peaks: (1) aloe-emodin; (2) rhein; (3) emodin; (4) chrysophanol; (5) physcion).

The optimized conditions were used for the extraction of target anthraquinones. As can be seen from Figure 7, by using BTO-SPE as sorbent, higher recoveries (85–96%) of anthraquinones than those of C18 sorbent (recoveries were in the range of 72–80%) were obtained. The higher recoveries by using BTO-SPE clearly demonstrated the worth of BTO-SPE as a new kind of SPE sorbent. In addition to this, C18 sorbent shows weaker matrix removing effect in the urine sample (Figure 8(B)). The outstanding enrichment and cleanup results of BTO-SPE sorbent maybe resulted from the mixed-mode characteristic and multiple-interactions with the target analytes. This unique properties make BTO-SPE material an available sorbent material to extract trace-level anthraquinones.

### 3.3. Method Validation

The optimized parameters for the extraction of anthraquinones compounds by using BTO-SPE as sorbents were as follows: treated urine sample (1 mL, pH 6) was loaded onto BTO-SPE sorbent (100 mg), the cartridge was washed by using 2 mL 5% MeOH solution, and then 2 mL MeOH was applied to elute the anthraquinones fraction; the eluate was collected and evaporated to dryness at 35°C under a gentle stream of N_2_ and then redissolved into 100 *μ*L 0.1% phosphoric acid water/methanol solution (50 : 50, v/v) prior to the UHPLC-FLD analysis. To evaluate the feasibility of the method developed, Figure 8 shows the HPLC chromatograms of blank and spiked human urine samples. All the urine samples were treated with the same SPE procedures prior to UHPLC analysis. No matrix effects, such as endogenous components or protein, were observed in urine sample chromatogram, indicating excellent specificity for the determination of anthraquinones in human urine.

Under the optimized conditions, the proposed method was investigated by evaluating the linear curves, limits of detection (LODs), and other characteristics. As shown in Table 1, all analytes exhibited good linearity from 0.012 to 1.800 *μ*g mL^−1^ with correlation coefficients (*r*^2^) ranging from 0.9993 to 0.9997. The LODs were in the range of 3.9–5.7 ng mL^−1^ based on a signal-to-noise ratio of 3, while the limits of quantification (LOQs) were in the range of 12.0–18.2 ng mL^−1^ based the ratio of signal-to-noise of 10. The relative standard deviations (RSDs) of peak area and retention time were in the range of 1.84–3.05% and 0.22–0.42%, respectively. These excellent results suggested that the proposed method was applicable to determine trace-level anthraquinones in human urine.

The intraday and interday precision of this SPE-UPLC-FLD method was investigated by testing three spiked urine samples at different concentration levels (20, 100, and 500 ng g^−1^) in accordance with the whole extraction process described above. The intraday precision of the assay was assessed by analyzing anthraquinones in spiked samples three replicates in the same day, while the interday precision was performed on three consecutive days using the optimized assay conditions. As shown in Table 2, the relative standard deviations (RSDs) of interday and intraday analysis were in the range of 2.85–4.58% and 2.03–2.94%, respectively. All these data are showing good feasibility as a bioanalytical method.

The recovery of the method was assessed by using blank urine spiked at concentrations of high, medium, and low concentrations for each analyte. The recoveries of anthraquinones were determined and shown in Table 2, which ranged from 94.8 to 106.6%. Thus, the developed UHPLC method based on BTO-SPE is suitable for analyzing the anthraquinones in biological samples.

### 3.4. Real Human Urine Sample Analysis

The proposed means were used for the determination of anthraquinones in urine from five patients with cerebral traumatic injury after oral rhubarb decoction. The typical chromatograms of human urine on BTO-SPE cartridge were shown in Figure 8, showing that anthraquinones obtained better separation from the sample matrixes. The contents of anthraquinones in the decoction were determined by UHPLC-FLD, which were 17.50 ± 0.94, 105.12 ± 2.14, 47.90 ± 1.39, 58.98 ± 1.26, and 29.15 ± 1.82 *μ*g mL^−1^ for chrysophanol, rhein, emodin, aloe-emodin, and physcion, respectively. The 36 h urine samples were collected after taking rhubarb decoction orally at the dosage of 0.05 g/kg. This experiment has been approved by the medical ethics committee of Henan University of Chinese Medicine, and the informed consent was signed before the experiment. Pretreatment of 1 mL urine sample was carried out according to the optimized procedure, the concentration of anthraquinones was analyzed, and the results are shown in Table 3. The recoveries of anthraquinones were determined by spiking the anthraquinones of 100 ng mL^−1^, and their recoveries ranged from 95.4 to 104.5%, indicating that the proposed UHPLC-FLD method based on BTO-SPE was appropriate for the analysis of anthraquinones in human urine.

The developed method was compared with the reported methods for the analysis of anthraquinones in biological or herbal medicine samples. As summarized in Table 4, the BTO-SPE-FLD method provided higher recoveries and lower LODs, than [11, 13, 17, 19], which was ascribed to the contribution of the mixed-mode and multiple-interactions BTO-SPE materials. In addition to that, the method developed also showed satisfactory linearity and equivalent or lower RSDs and LODs values.

## 4. Conclusion

In this study, a UHPLC-FLD method has been proposed for the simultaneous analysis of five trace-level anthraquinones in human urine by using BTO-SPE as sorbent. With the optimal sample pretreatment condition, the method was successfully applied to urine samples from 5 patients. Comparative study showed BTO-SPE sorbent had excellent cleanup and enrichment property of anthraquinones due to its multi-interaction ability. The extraction process probably was regulated by multiple-interactions, including hydrophobic, hydrogen bonding, dipole–dipole, and *π*-*π* interactions. The proposed UHPLC-FLD method based on BTO-SPE is promising for the determination of anthraquinones in biological samples.

## Figures and Tables

**Table 1:** Analytical performance data of the proposed method.

Analyte	Calibration curves	*r* ^2^	Linear range	LOD	LOQ	RSD (%, *n* = 3)	
			*μ*g mL^−1^	ng mL^−1^	ng mL^−1^	Peak area	Retention time
Aloe-emodin	*y* = 10.67*x* − 0.012	0.9997	0.012–1.200	4.1	12.1	2.35	0.38
Rhein	*y* = 9.14*x* − 0.020	0.9995	0.018–1.800	5.7	18.2	2.21	0.42
Emodin	*y* = 9.66*x* − 0.011	0.9996	0.015–1.500	5.1	15.2	1.84	0.29
Chrysophanol	*y* = 10.48*x* − 0.016	0.9993	0.012–1.200	3.9	12.0	2.48	0.26
Physcion	*y* = 9.55*x* − 0.012	0.9996	0.013–1.300	4.4	13.1	3.05	0.22

**Table 2:** The interday and intraday precision and recoveries of the method.

Analytes	Spiked amount (ng/g)	Intraday (*n* = 3)		Interday (*n* = 3)	
		Recovery (%)	RSD (%)	Recovery (%)	RSD (%)
Aloe-emodin	20	97.3	2.26	96.7	2.88
	100	96.8	2.58	97.5	3.15
	500	95.2	2.42	94.8	3.36

Rhein	20	104.3	2.03	102.8	3.28
	100	97.5	2.83	98.1	2.95
	500	96.3	2.78	97.4	3.45

Emodin	20	105.6	2.75	106.6	3.47
	100	96.7	2.63	95.8	4.58
	500	94.9	2.86	95.4	3.48

Chrysophanol	20	97.7	2.05	98.0	2.85
	100	98.3	2.83	97.7	3.15
	500	95.8	2.75	96.2	3.42

Physcion	20	104.6	2.46	105.6	4.03
	100	95.8	2.94	96.3	3.85
	500	96.8	2.81	96.4	3.07

**Table 3:** Determination of anthraquinones in urine from five patients after oral rhubarb decoction.

Sample number	Aloe-emodin	Rhein	Emodin	Chrysophanol	Physcion
	*μ*g mL^−1^				
(1)	0.078	0.662	0.047	0.152	0.038
(2)	0.054	0.782	0.032	0.236	0.027
(3)	0.068	0.715	0.038	0.132	0.038
(4)	0.062	0.813	0.071	0.086	0.034
(5)	0.085	0.764	0.058	0.124	0.056

**Table 4:** Comparison of BTO-SPE-FLD method with other methods for the determination of anthraquinones.

Clean-up	Determination technique	Linearity (*μ*g mL^−1^)	Recoveries (%)	RSD (%)	LOD (*μ*g mL^−1^)	References
HLB-SPE	MEKC^a^	5–50	98–107	<2.55	0.50–0.58	[11]
C18-SPE	HPLC-FLD	0.0210–19.52	94.2–110.4	<7.4	0.007–0.0133	[13]
MIP-MSPD^b^	HPLC-UV	1–200	91.2–101.4	5.3–7.1	0.23–0.28	[17]
Soxhlet extraction	CD-MEKC^c^	3.86–85	91.1–100.08	1.12–2.01	0.75–1.15	[19]
BTO-SPE	UHPLC-FLD	0.012–1.8	94.9–105.6	2.03–2.86	0.0039–0.0057	This method

^a^micellar electrokinetic chromatography; ^b^molecularly imprinted polymer-matrix solid-phase dispersion; ^c^cyclodextrin-modified micellar electrokinetic chromatography.
